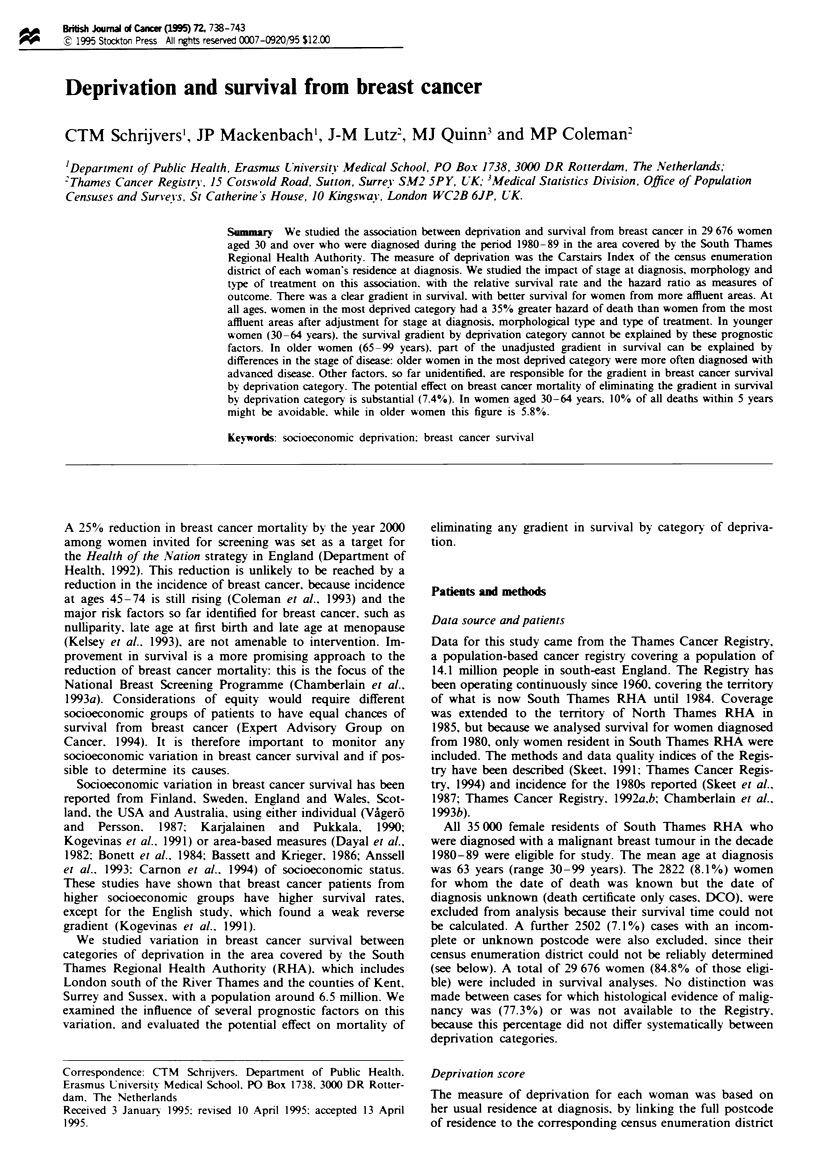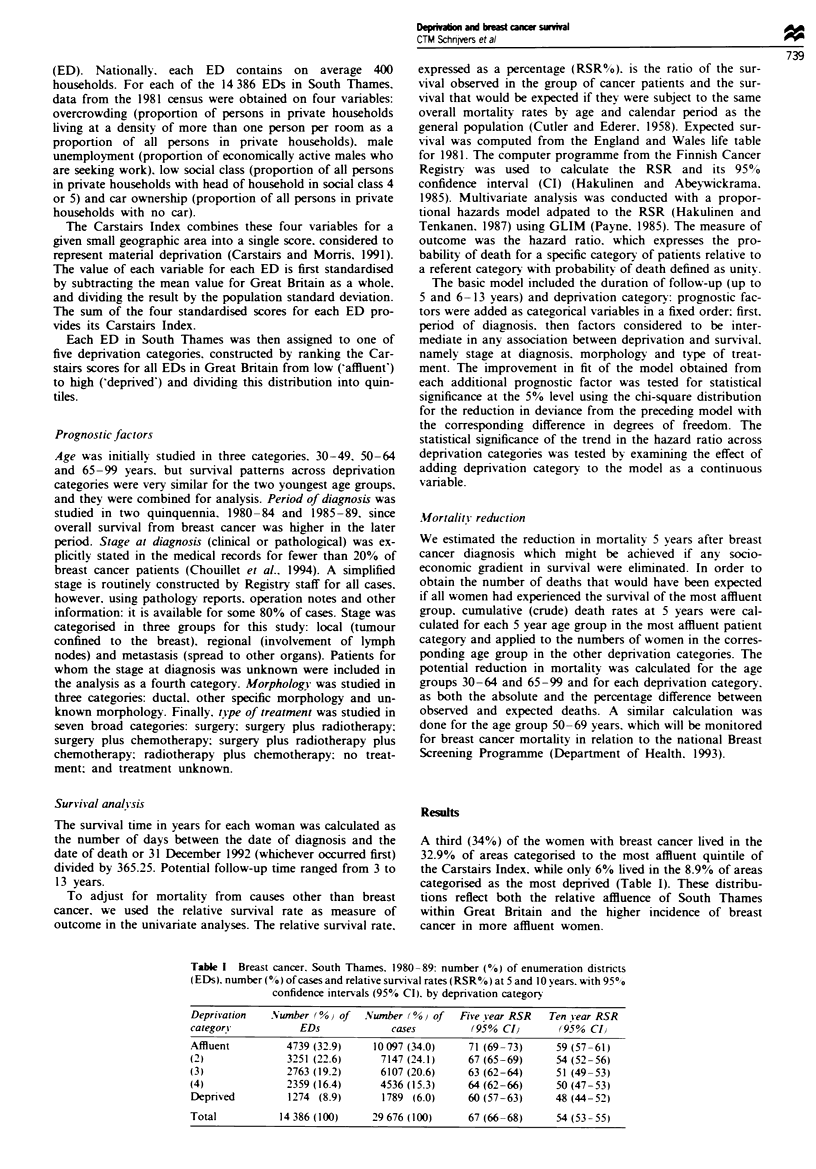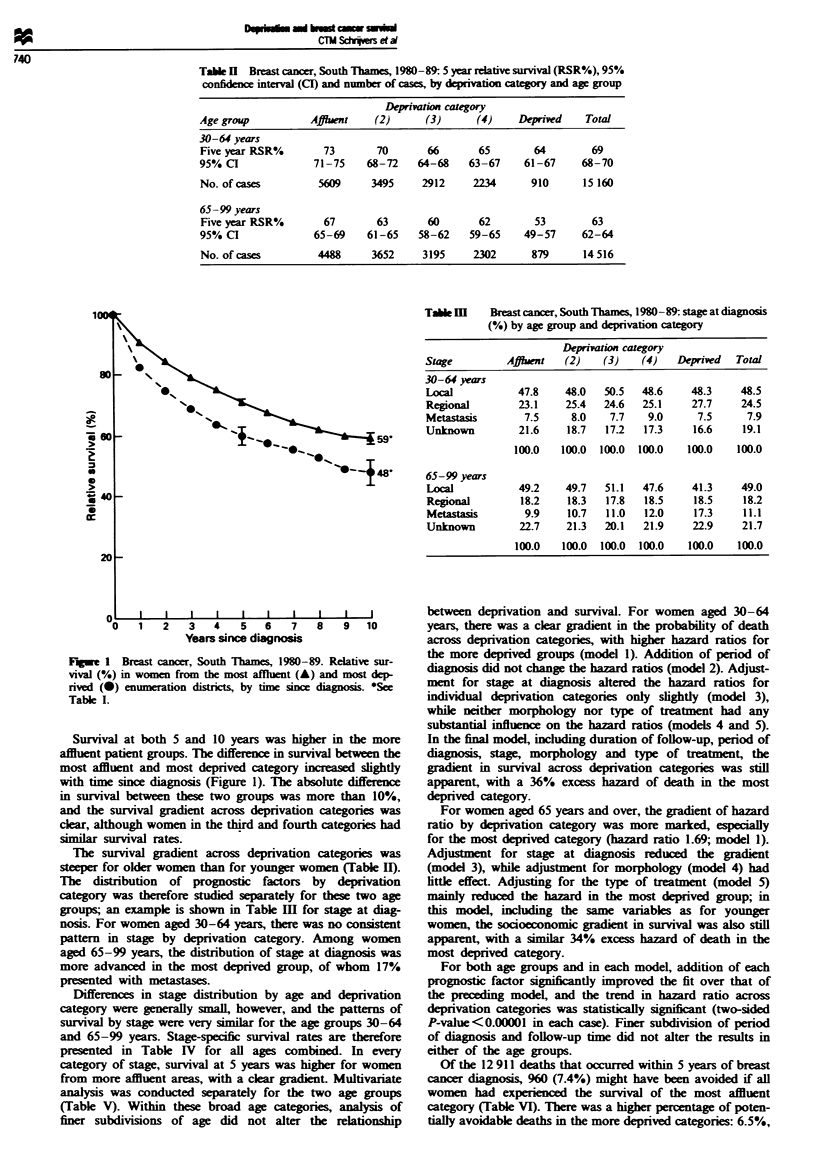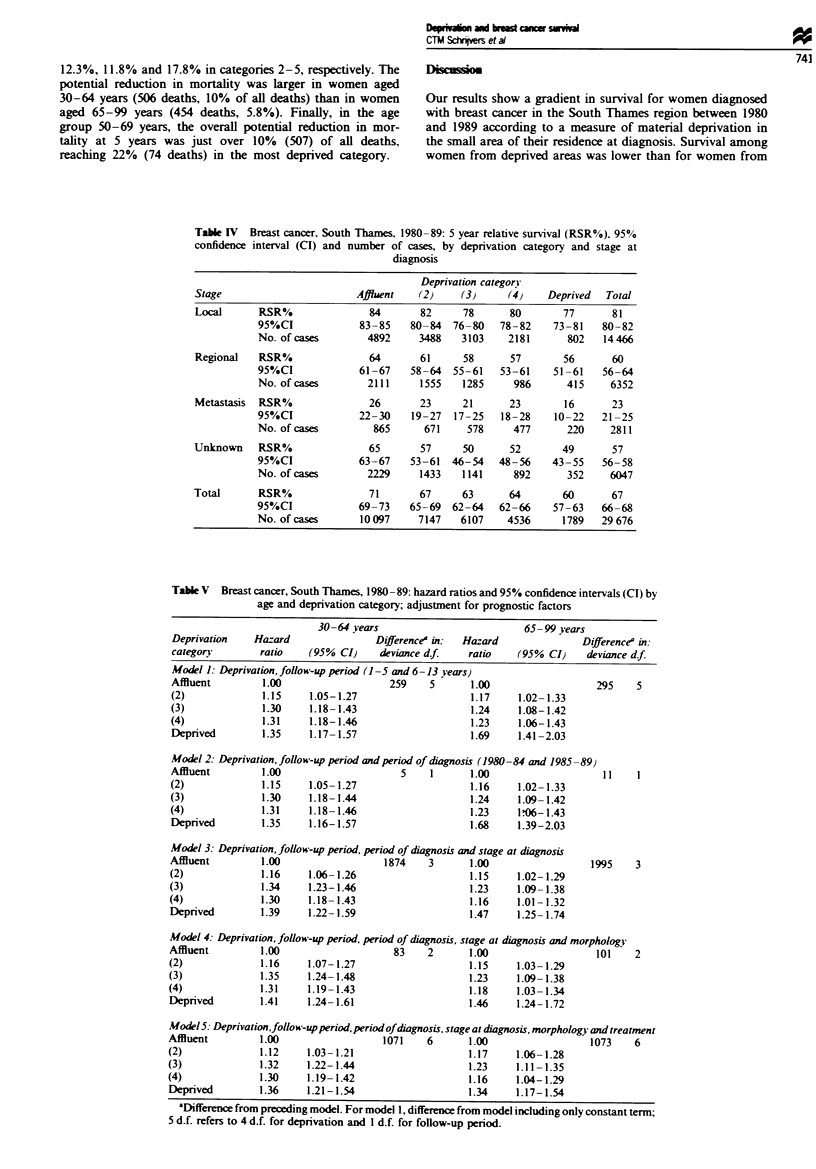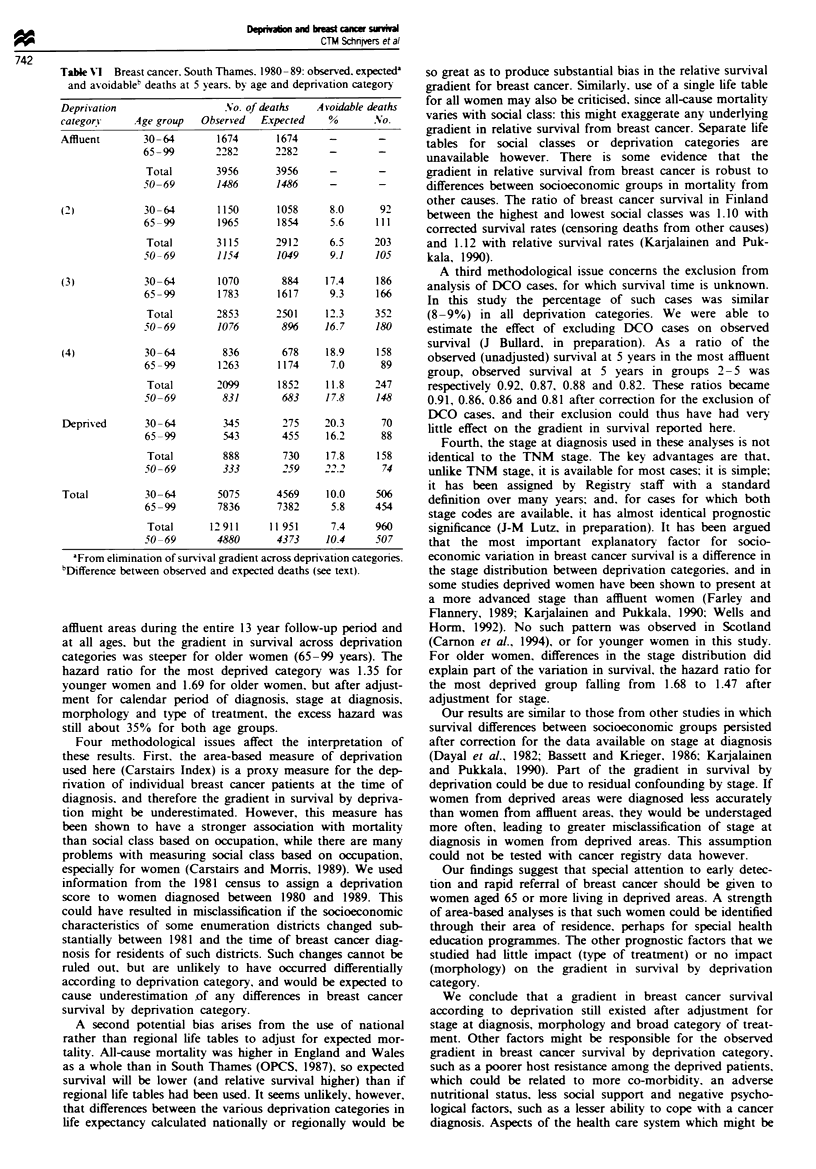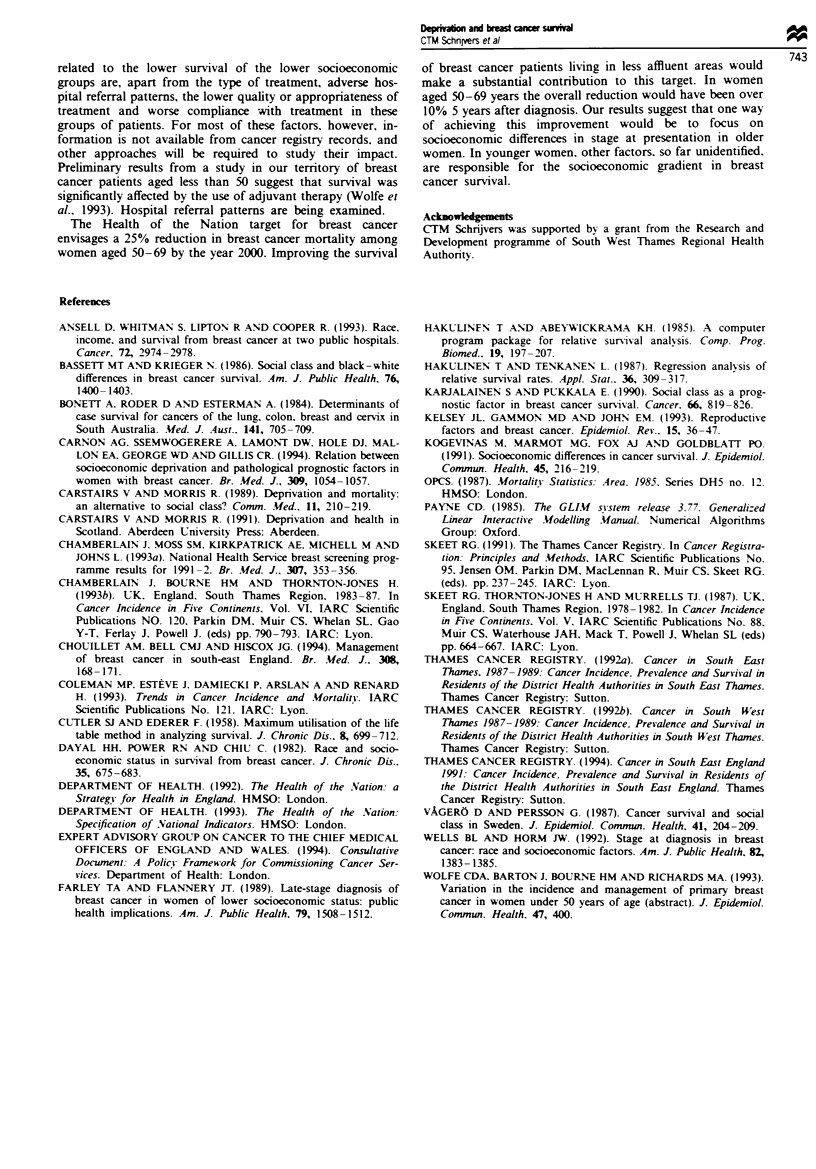# Deprivation and survival from breast cancer.

**DOI:** 10.1038/bjc.1995.403

**Published:** 1995-09

**Authors:** C. T. Schrijvers, J. P. Mackenbach, J. M. Lutz, M. J. Quinn, M. P. Coleman

**Affiliations:** Department of Public Health, Erasmus University Medical School, Rotterdam, The Netherlands.

## Abstract

We studied the association between deprivation and survival from breast cancer in 29,676 women aged 30 and over who were diagnosed during the period 1980-89 in the area covered by the South Thames Regional Health Authority. The measure of deprivation was the Carstairs Index of the census enumeration district of each woman's residence at diagnosis. We studied the impact of stage at diagnosis, morphology and type of treatment on this association, with the relative survival rate and the hazard ratio as measures of outcome. There was a clear gradient in survival, with better survival for women from more affluent areas. At all ages, women in the most deprived category had a 35% greater hazard of death than women from the most affluent areas after adjustment for stage at diagnosis, morphological type and type of treatment. In younger women (30-64 years), the survival gradient by deprivation category cannot be explained by these prognostic factors. In older women (65-99 years), part of the unadjusted gradient in survival can be explained by differences in the stage of disease: older women in the most deprived category were more often diagnosed with advanced disease. Other factors, so far unidentified, are responsible for the gradient in breast cancer survival by deprivation category. The potential effect on breast cancer mortality of eliminating the gradient in survival by deprivation category is substantial (7.4%). In women aged 30-64 years, 10% of all deaths within 5 years might be avoidable, while in older women this figure is 5.8%.


British Journal of Cancer (1995) 72, 738-743

© 1995 Stockton Press All rights reserved 0007-0920/95 $12.00

### Deprivation and survival from breast cancer

CTM Schrijvers[1], JP Mackenbach[1], J-M Lutz[2], MJ Quinn[3] and MP Coleman[2]

[1]*Department of Public Health, Erasmus University Medical School, PO Box 1738, 3000 DR Rotterdam, The Netherlands;* [2]*Thames Cancer Registry, 15 Cotswold Road, Sutton, Surrey SM2 5PY, UK;* [3]*Medical Statistics Division, Office of Population Censuses and Surveys, St Catherine's House, 10 Kingsway, London WC2B 6JP, UK.*

**Summary** We studied the association between deprivation and survival from breast cancer in 29 676 women aged 30 and over who were diagnosed during the period 1980-89 in the area covered by the South Thames Regional Health Authority. The measure of deprivation was the Carstairs Index of the census enumeration district of each woman's residence at diagnosis. We studied the impact of stage at diagnosis, morphology and type of treatment on this association, with the relative survival rate and the hazard ratio as measures of outcome. There was a clear gradient in survival, with better survival for women from more affluent areas. At all ages, women in the most deprived category had a 35% greater hazard of death than women from the most affluent areas after adjustment for stage at diagnosis, morphological type and type of treatment. In younger women (30-64 years), the survival gradient by deprivation category cannot be explained by these prognostic factors. In older women (65-99 years), part of the unadjusted gradient in survival can be explained by differences in the stage of disease: older women in the most deprived category were more often diagnosed with advanced disease. Other factors, so far unidentified, are responsible for the gradient in breast cancer survival by deprivation category. The potential effect on breast cancer mortality of eliminating the gradient in survival by deprivation category is substantial (7.4%). In women aged 30-64 years, 10% of all deaths within 5 years might be avoidable, while in older women this figure is 5.8%.

**Keywords**: socioeconomic deprivation; breast cancer survival

A 25% reduction in breast cancer mortality by the year 2000 among women invited for screening was set as a target for the *Health of the Nation* strategy in England (Department of Health, 1992). This reduction is unlikely to be reached by a reduction in the incidence of breast cancer, because incidence at ages 45-74 is still rising (Coleman *et al.*, 1993) and the major risk factors so far identified for breast cancer, such as nulliparity, late age at first birth and late age at menopause (Kelsey *et al.*, 1993), are not amenable to intervention. Improvement in survival is a more promising approach to the reduction of breast cancer mortality: this is the focus of the National Breast Screening Programme (Chamberlain *et al.*, 1993*a*). Considerations of equity would require different socioeconomic groups of patients to have equal chances of survival from breast cancer (Expert Advisory Group on Cancer, 1994). It is therefore important to monitor any socioeconomic variation in breast cancer survival and if possible to determine its causes.

Socioeconomic variation in breast cancer survival has been reported from Finland, Sweden, England and Wales, Scotland, the USA and Australia, using either individual (Vågerö and Persson, 1987; Karjalainen and Pukkala, 1990; Kogevinas *et al.*, 1991) or area-based measures (Dayal *et al.*, 1982; Bonett *et al.*, 1984; Bassett and Krieger, 1986; Anssell *et al.*, 1993; Carnon *et al.*, 1994) of socioeconomic status. These studies have shown that breast cancer patients from higher socioeconomic groups have higher survival rates, except for the English study, which found a weak reverse gradient (Kogevinas *et al.*, 1991).

We studied variation in breast cancer survival between categories of deprivation in the area covered by the South Thames Regional Health Authority (RHA), which includes London south of the River Thames and the counties of Kent, Surrey and Sussex, with a population around 6.5 million. We examined the influence of several prognostic factors on this variation, and evaluated the potential effect on mortality of eliminating any gradient in survival by category of deprivation.

Correspondence: CTM Schrijvers, Department of Public Health, Erasmus University Medical School, PO Box 1738, 3000 DR Rotterdam, The Netherlands
Received 3 January 1995; revised 10 April 1995; accepted 13 April 1995.

### Patients and methods

### *Data source and patients*

Data for this study came from the Thames Cancer Registry, a population-based cancer registry covering a population of 14.1 million people in south-east England. The Registry has been operating continuously since 1960, covering the territory of what is now South Thames RHA until 1984. Coverage was extended to the territory of North Thames RHA in 1985, but because we analysed survival for women diagnosed from 1980, only women resident in South Thames RHA were included. The methods and data quality indices of the Registry have been described (Skeet, 1991; Thames Cancer Registry, 1994) and incidence for the 1980s reported (Skeet *et al.*, 1987; Thames Cancer Registry, 1992*a*,*b*; Chamberlain *et al.*, 1993*b*).

All 35 000 female residents of South Thames RHA who were diagnosed with a malignant breast tumour in the decade 1980-89 were eligible for study. The mean age at diagnosis was 63 years (range 30-99 years). The 2822 (8.1%) women for whom the date of death was known but the date of diagnosis unknown (death certificate only cases, DCO), were excluded from analysis because their survival time could not be calculated. A further 2502 (7.1%) cases with an incomplete or unknown postcode were also excluded, since their census enumeration district could not be reliably determined (see below). A total of 29 676 women (84.8% of those eligible) were included in survival analyses. No distinction was made between cases for which histological evidence of malignancy was (77.3%) or was not available to the Registry, because this percentage did not differ systematically between deprivation categories.

### *Deprivation score*

The measure of deprivation for each woman was based on her usual residence at diagnosis, by linking the full postcode of residence to the corresponding census enumeration district

(ED). Nationally, each ED contains on average 400 households. For each of the 14 386 EDs in South Thames, data from the 1981 census were obtained on four variables: overcrowding (proportion of persons in private households living at a density of more than one person per room as a proportion of all persons in private households), male unemployment (proportion of economically active males who are seeking work), low social class (proportion of all persons in private households with head of household in social class 4 or 5) and car ownership (proportion of all persons in private households with no car).

The Carstairs Index combines these four variables for a given small geographic area into a single score, considered to represent material deprivation (Carstairs and Morris, 1991). The value of each variable for each ED is first standardised by subtracting the mean value for Great Britain as a whole, and dividing the result by the population standard deviation. The sum of the four standardised scores for each ED provides its Carstairs Index.

Each ED in South Thames was then assigned to one of five deprivation categories, constructed by ranking the Carstairs scores for all EDs in Great Britain from low ('affluent') to high ('deprived') and dividing this distribution into quintiles.

### *Prognostic factors*

*Age* was initially studied in three categories, 30–49, 50–64 and 65–99 years, but survival patterns across deprivation categories were very similar for the two youngest age groups, and they were combined for analysis. *Period of diagnosis* was studied in two quinquennia, 1980–84 and 1985–89, since overall survival from breast cancer was higher in the later period. *Stage at diagnosis* (clinical or pathological) was explicitly stated in the medical records for fewer than 20% of breast cancer patients (Chouillet *et al*., 1994). A simplified stage is routinely constructed by Registry staff for all cases, however, using pathology reports, operation notes and other information: it is available for some 80% of cases. Stage was categorised in three groups for this study: local (tumour confined to the breast), regional (involvement of lymph nodes) and metastasis (spread to other organs). Patients for whom the stage at diagnosis was unknown were included in the analysis as a fourth category. *Morphology* was studied in three categories: ductal, other specific morphology and unknown morphology. Finally, *type of treatment* was studied in seven broad categories: surgery; surgery plus radiotherapy; surgery plus chemotherapy; surgery plus radiotherapy plus chemotherapy; radiotherapy plus chemotherapy; no treatment; and treatment unknown.

### *Survival analysis*

The survival time in years for each woman was calculated as the number of days between the date of diagnosis and the date of death or 31 December 1992 (whichever occurred first) divided by 365.25. Potential follow-up time ranged from 3 to 13 years.

To adjust for mortality from causes other than breast cancer, we used the relative survival rate as measure of outcome in the univariate analyses. The relative survival rate, expressed as a percentage (RSR%), is the ratio of the survival observed in the group of cancer patients and the survival that would be expected if they were subject to the same overall mortality rates by age and calendar period as the general population (Cutler and Ederer, 1958). Expected survival was computed from the England and Wales life table for 1981. The computer programme from the Finnish Cancer Registry was used to calculate the RSR and its 95% confidence interval (CI) (Hakulinen and Abeywickrama, 1985). Multivariate analysis was conducted with a proportional hazards model adpated to the RSR (Hakulinen and Tenkanen, 1987) using GLIM (Payne, 1985). The measure of outcome was the hazard ratio, which expresses the probability of death for a specific category of patients relative to a referent category with probability of death defined as unity.

The basic model included the duration of follow-up (up to 5 and 6–13 years) and deprivation category; prognostic factors were added as categorical variables in a fixed order: first, period of diagnosis, then factors considered to be intermediate in any association between deprivation and survival, namely stage at diagnosis, morphology and type of treatment. The improvement in fit of the model obtained from each additional prognostic factor was tested for statistical significance at the 5% level using the chi-square distribution for the reduction in deviance from the preceding model with the corresponding difference in degrees of freedom. The statistical significance of the trend in the hazard ratio across deprivation categories was tested by examining the effect of adding deprivation category to the model as a continuous variable.

### *Mortality reduction*

We estimated the reduction in mortality 5 years after breast cancer diagnosis which might be achieved if any socioeconomic gradient in survival were eliminated. In order to obtain the number of deaths that would have been expected if all women had experienced the survival of the most affluent group, cumulative (crude) death rates at 5 years were calculated for each 5 year age group in the most affluent patient category and applied to the numbers of women in the corresponding age group in the other deprivation categories. The potential reduction in mortality was calculated for the age groups 30–64 and 65–99 and for each deprivation category, as both the absolute and the percentage difference between observed and expected deaths. A similar calculation was done for the age group 50–69 years, which will be monitored for breast cancer mortality in relation to the national Breast Screening Programme (Department of Health, 1993).

### Results

A third (34%) of the women with breast cancer lived in the 32.9% of areas categorised to the most affluent quintile of the Carstairs Index, while only 6% lived in the 8.9% of areas categorised as the most deprived (Table I). These distributions reflect both the relative affluence of South Thames within Great Britain and the higher incidence of breast cancer in more affluent women.

**Table I** Breast cancer, South Thames, 1980–89: number (%) of enumeration districts (EDs), number (%) of cases and relative survival rates (RSR%) at 5 and 10 years, with 95% confidence intervals (95% CI), by deprivation category

| *Deprivation category* | *Number (%) of EDs* | *Number (%) of cases* | *Five year RSR (95% CI)* | *Ten year RSR (95% CI)* |
|---|---|---|---|---|
| Affluent | 4739 (32.9) | 10 097 (34.0) | 71 (69–73) | 59 (57–61) |
| (2) | 3251 (22.6) | 7147 (24.1) | 67 (65–69) | 54 (52–56) |
| (3) | 2763 (19.2) | 6107 (20.6) | 63 (62–64) | 51 (49–53) |
| (4) | 2359 (16.4) | 4536 (15.3) | 64 (62–66) | 50 (47–53) |
| Deprived | 1274 (8.9) | 1789 (6.0) | 60 (57–63) | 48 (44–52) |
| Total | 14 386 (100) | 29 676 (100) | 67 (66–68) | 54 (53–55) |

**Table II** Breast cancer, South Thames, 1980–89: 5 year relative survival (RSR%), 95% confidence interval (CI) and number of cases, by deprivation category and age group

| | Deprivation category | | | | | |
|---|---|---|---|---|---|---|
| *Age group* | *Affluent* | *(2)* | *(3)* | *(4)* | *Deprived* | *Total* |
| *30–64 years* | | | | | | |
| Five year RSR% | 73 | 70 | 66 | 65 | 64 | 69 |
| 95% CI | 71–75 | 68–72 | 64–68 | 63–67 | 61–67 | 68–70 |
| No. of cases | 5609 | 3495 | 2912 | 2234 | 910 | 15 160 |
| *65–99 years* | | | | | | |
| Five year RSR% | 67 | 63 | 60 | 62 | 53 | 63 |
| 95% CI | 65–69 | 61–65 | 58–62 | 59–65 | 49–57 | 62–64 |
| No. of cases | 4488 | 3652 | 3195 | 2302 | 879 | 14 516 |

**Figure 1** Breast cancer, South Thames, 1980–89. Relative survival (%) in women from the most affluent (▲) and most deprived (●) enumeration districts, by time since diagnosis. *See Table I.

**Table III** Breast cancer, South Thames, 1980–89: stage at diagnosis (%) by age group and deprivation category

| | Deprivation category | | | | | |
|---|---|---|---|---|---|---|
| *Stage* | *Affluent* | *(2)* | *(3)* | *(4)* | *Deprived* | *Total* |
| *30–64 years* | | | | | | |
| Local | 47.8 | 48.0 | 50.5 | 48.6 | 48.3 | 48.5 |
| Regional | 23.1 | 25.4 | 24.6 | 25.1 | 27.7 | 24.5 |
| Metastasis | 7.5 | 8.0 | 7.7 | 9.0 | 7.5 | 7.9 |
| Unknown | 21.6 | 18.7 | 17.2 | 17.3 | 16.6 | 19.1 |
| | 100.0 | 100.0 | 100.0 | 100.0 | 100.0 | 100.0 |
| *65–99 years* | | | | | | |
| Local | 49.2 | 49.7 | 51.1 | 47.6 | 41.3 | 49.0 |
| Regional | 18.2 | 18.3 | 17.8 | 18.5 | 18.5 | 18.2 |
| Metastasis | 9.9 | 10.7 | 11.0 | 12.0 | 17.3 | 11.1 |
| Unknown | 22.7 | 21.3 | 20.1 | 21.9 | 22.9 | 21.7 |
| | 100.0 | 100.0 | 100.0 | 100.0 | 100.0 | 100.0 |

Survival at both 5 and 10 years was higher in the more affluent patient groups. The difference in survival between the most affluent and most deprived category increased slightly with time since diagnosis (Figure 1). The absolute difference in survival between these two groups was more than 10%, and the survival gradient across deprivation categories was clear, although women in the third and fourth categories had similar survival rates.

The survival gradient across deprivation categories was steeper for older women than for younger women (Table II). The distribution of prognostic factors by deprivation category was therefore studied separately for these two age groups; an example is shown in Table III for stage at diagnosis. For women aged 30–64 years, there was no consistent pattern in stage by deprivation category. Among women aged 65–99 years, the distribution of stage at diagnosis was more advanced in the most deprived group, of whom 17% presented with metastases.

Differences in stage distribution by age and deprivation category were generally small, however, and the patterns of survival by stage were very similar for the age groups 30–64 and 65–99 years. Stage-specific survival rates are therefore presented in Table IV for all ages combined. In every category of stage, survival at 5 years was higher for women from more affluent areas, with a clear gradient. Multivariate analysis was conducted separately for the two age groups (Table V). Within these broad age categories, analysis of finer subdivisions of age did not alter the relationship between deprivation and survival. For women aged 30–64 years, there was a clear gradient in the probability of death across deprivation categories, with higher hazard ratios for the more deprived groups (model 1). Addition of period of diagnosis did not change the hazard ratios (model 2). Adjustment for stage at diagnosis altered the hazard ratios for individual deprivation categories only slightly (model 3), while neither morphology nor type of treatment had any substantial influence on the hazard ratios (models 4 and 5). In the final model, including duration of follow-up, period of diagnosis, stage, morphology and type of treatment, the gradient in survival across deprivation categories was still apparent, with a 36% excess hazard of death in the most deprived category.

For women aged 65 years and over, the gradient of hazard ratio by deprivation category was more marked, especially for the most deprived category (hazard ratio 1.69; model 1). Adjustment for stage at diagnosis reduced the gradient (model 3), while adjustment for morphology (model 4) had little effect. Adjusting for the type of treatment (model 5) mainly reduced the hazard in the most deprived group; in this model, including the same variables as for younger women, the socioeconomic gradient in survival was also still apparent, with a similar 34% excess hazard of death in the most deprived category.

For both age groups and in each model, addition of each prognostic factor significantly improved the fit over that of the preceding model, and the trend in hazard ratio across deprivation categories was statistically significant (two-sided $P$-value $<0.00001$ in each case). Finer subdivision of period of diagnosis and follow-up time did not alter the results in either of the age groups.

Of the 12 911 deaths that occurred within 5 years of breast cancer diagnosis, 960 (7.4%) might have been avoided if all women had experienced the survival of the most affluent category (Table VI). There was a higher percentage of potentially avoidable deaths in the more deprived categories: 6.5%,

12.3%, 11.8% and 17.8% in categories 2–5, respectively. The potential reduction in mortality was larger in women aged 30–64 years (506 deaths, 10% of all deaths) than in women aged 65–99 years (454 deaths, 5.8%). Finally, in the age group 50–69 years, the overall potential reduction in mortality at 5 years was just over 10% (507) of all deaths, reaching 22% (74 deaths) in the most deprived category.

### Discussion

Our results show a gradient in survival for women diagnosed with breast cancer in the South Thames region between 1980 and 1989 according to a measure of material deprivation in the small area of their residence at diagnosis. Survival among women from deprived areas was lower than for women from

**Table IV** Breast cancer, South Thames, 1980–89: 5 year relative survival (RSR%), 95% confidence interval (CI) and number of cases, by deprivation category and stage at diagnosis

| *Stage* | | *Affluent* | *Deprivation category* (2) | (3) | (4) | *Deprived* | *Total* |
|---|---|---|---|---|---|---|---|
| Local | RSR% | 84 | 82 | 78 | 80 | 77 | 81 |
| | 95%CI | 83–85 | 80–84 | 76–80 | 78–82 | 73–81 | 80–82 |
| | No. of cases | 4892 | 3488 | 3103 | 2181 | 802 | 14 466 |
| Regional | RSR% | 64 | 61 | 58 | 57 | 56 | 60 |
| | 95%CI | 61–67 | 58–64 | 55–61 | 53–61 | 51–61 | 56–64 |
| | No. of cases | 2111 | 1555 | 1285 | 986 | 415 | 6352 |
| Metastasis | RSR% | 26 | 23 | 21 | 23 | 16 | 23 |
| | 95%CI | 22–30 | 19–27 | 17–25 | 18–28 | 10–22 | 21–25 |
| | No. of cases | 865 | 671 | 578 | 477 | 220 | 2811 |
| Unknown | RSR% | 65 | 57 | 50 | 52 | 49 | 57 |
| | 95%CI | 63-67 | 53-61 | 46-54 | 48-56 | 43-55 | 56-58 |
| | No. of cases | 2229 | 1433 | 1141 | 892 | 352 | 6047 |
| Total | RSR% | 71 | 67 | 63 | 64 | 60 | 67 |
| | 95%CI | 69–73 | 65–69 | 62–64 | 62–66 | 57–63 | 66–68 |
| | No. of cases | 10 097 | 7147 | 6107 | 4536 | 1789 | 29 676 |

**Table V** Breast cancer, South Thames, 1980–89: hazard ratios and 95% confidence intervals (CI) by age and deprivation category; adjustment for prognostic factors

| *Deprivation category* | *30–64 years* *Hazard ratio* | *(95% CI)* | *Difference[a] in: deviance* | *d.f.* | *65–99 years* *Hazard ratio* | *(95% CI)* | *Difference[a] in: deviance* | *d.f.* |
|---|---|---|---|---|---|---|---|---|
| *Model 1: Deprivation, follow-up period (1–5 and 6–13 years)* | | | | | | | | |
| Affluent | 1.00 | | 259 | 5 | 1.00 | | 295 | 5 |
| (2) | 1.15 | 1.05–1.27 | | | 1.17 | 1.02–1.33 | | |
| (3) | 1.30 | 1.18–1.43 | | | 1.24 | 1.08–1.42 | | |
| (4) | 1.31 | 1.18–1.46 | | | 1.23 | 1.06–1.43 | | |
| Deprived | 1.35 | 1.17–1.57 | | | 1.69 | 1.41–2.03 | | |
| *Model 2: Deprivation, follow-up period and period of diagnosis (1980–84 and 1985–89)* | | | | | | | | |
| Affluent | 1.00 | | 5 | 1 | 1.00 | | 11 | 1 |
| (2) | 1.15 | 1.05–1.27 | | | 1.16 | 1.02–1.33 | | |
| (3) | 1.30 | 1.18–1.44 | | | 1.24 | 1.09–1.42 | | |
| (4) | 1.31 | 1.18–1.46 | | | 1.23 | 1.06–1.43 | | |
| Deprived | 1.35 | 1.16–1.57 | | | 1.68 | 1.39–2.03 | | |
| *Model 3: Deprivation, follow-up period, period of diagnosis and stage at diagnosis* | | | | | | | | |
| Affluent | 1.00 | | 1874 | 3 | 1.00 | | 1995 | 3 |
| (2) | 1.16 | 1.06–1.26 | | | 1.15 | 1.02–1.29 | | |
| (3) | 1.34 | 1.23–1.46 | | | 1.23 | 1.09–1.38 | | |
| (4) | 1.30 | 1.18–1.43 | | | 1.16 | 1.01–1.32 | | |
| Deprived | 1.39 | 1.22–1.59 | | | 1.47 | 1.25–1.74 | | |
| *Model 4: Deprivation, follow-up period, period of diagnosis, stage at diagnosis and morphology* | | | | | | | | |
| Affluent | 1.00 | | 83 | 2 | 1.00 | | 101 | 2 |
| (2) | 1.16 | 1.07–1.27 | | | 1.15 | 1.03–1.29 | | |
| (3) | 1.35 | 1.24–1.48 | | | 1.23 | 1.09–1.38 | | |
| (4) | 1.31 | 1.19–1.43 | | | 1.18 | 1.03–1.34 | | |
| Deprived | 1.41 | 1.24–1.61 | | | 1.46 | 1.24–1.72 | | |
| *Model 5: Deprivation, follow-up period, period of diagnosis, stage at diagnosis, morphology and treatment* | | | | | | | | |
| Affluent | 1.00 | | 1071 | 6 | 1.00 | | 1073 | 6 |
| (2) | 1.12 | 1.03–1.21 | | | 1.17 | 1.06–1.28 | | |
| (3) | 1.32 | 1.22–1.44 | | | 1.23 | 1.11–1.35 | | |
| (4) | 1.30 | 1.19–1.42 | | | 1.16 | 1.04–1.29 | | |
| Deprived | 1.36 | 1.21–1.54 | | | 1.34 | 1.17–1.54 | | |

[a]Difference from preceding model. For model 1, difference from model including only constant term; 5 d.f. refers to 4 d.f. for deprivation and 1 d.f. for follow-up period.

**Table VI** Breast cancer, South Thames, 1980–89: observed, expected[a] and avoidable[b] deaths at 5 years, by age and deprivation category

| Deprivation category | Age group | No. of deaths Observed | Expected | Avoidable deaths % | No. |
|---|---|---|---|---|---|
| Affluent | 30–64 | 1674 | 1674 | – | – |
| | 65–99 | 2282 | 2282 | – | – |
| | Total | 3956 | 3956 | – | – |
| | *50–69* | *1486* | *1486* | – | – |
| (2) | 30–64 | 1150 | 1058 | 8.0 | 92 |
| | 65–99 | 1965 | 1854 | 5.6 | 111 |
| | Total | 3115 | 2912 | 6.5 | 203 |
| | *50–69* | *1154* | *1049* | *9.1* | *105* |
| (3) | 30–64 | 1070 | 884 | 17.4 | 186 |
| | 65–99 | 1783 | 1617 | 9.3 | 166 |
| | Total | 2853 | 2501 | 12.3 | 352 |
| | *50–69* | *1076* | *896* | *16.7* | *180* |
| (4) | 30–64 | 836 | 678 | 18.9 | 158 |
| | 65–99 | 1263 | 1174 | 7.0 | 89 |
| | Total | 2099 | 1852 | 11.8 | 247 |
| | *50–69* | *831* | *683* | *17.8* | *148* |
| Deprived | 30–64 | 345 | 275 | 20.3 | 70 |
| | 65–99 | 543 | 455 | 16.2 | 88 |
| | Total | 888 | 730 | 17.8 | 158 |
| | *50–69* | *333* | *259* | *22.2* | *74* |
| Total | 30–64 | 5075 | 4569 | 10.0 | 506 |
| | 65–99 | 7836 | 7382 | 5.8 | 454 |
| | Total | 12 911 | 11 951 | 7.4 | 960 |
| | *50–69* | *4880* | *4373* | *10.4* | *507* |

[a]From elimination of survival gradient across deprivation categories. [b]Difference between observed and expected deaths (see text).

affluent areas during the entire 13 year follow-up period and at all ages, but the gradient in survival across deprivation categories was steeper for older women (65–99 years). The hazard ratio for the most deprived category was 1.35 for younger women and 1.69 for older women, but after adjustment for calendar period of diagnosis, stage at diagnosis, morphology and type of treatment, the excess hazard was still about 35% for both age groups.

Four methodological issues affect the interpretation of these results. First, the area-based measure of deprivation used here (Carstairs Index) is a proxy measure for the deprivation of individual breast cancer patients at the time of diagnosis, and therefore the gradient in survival by deprivation might be underestimated. However, this measure has been shown to have a stronger association with mortality than social class based on occupation, while there are many problems with measuring social class based on occupation, especially for women (Carstairs and Morris, 1989). We used information from the 1981 census to assign a deprivation score to women diagnosed between 1980 and 1989. This could have resulted in misclassification if the socioeconomic characteristics of some enumeration districts changed substantially between 1981 and the time of breast cancer diagnosis for residents of such districts. Such changes cannot be ruled out, but are unlikely to have occurred differentially according to deprivation category, and would be expected to cause underestimation of any differences in breast cancer survival by deprivation category.

A second potential bias arises from the use of national rather than regional life tables to adjust for expected mortality. All-cause mortality was higher in England and Wales as a whole than in South Thames (OPCS, 1987), so expected survival will be lower (and relative survival higher) than if regional life tables had been used. It seems unlikely, however, that differences between the various deprivation categories in life expectancy calculated nationally or regionally would be so great as to produce substantial bias in the relative survival gradient for breast cancer. Similarly, use of a single life table for all women may also be criticised, since all-cause mortality varies with social class: this might exaggerate any underlying gradient in relative survival from breast cancer. Separate life tables for social classes or deprivation categories are unavailable however. There is some evidence that the gradient in relative survival from breast cancer is robust to differences between socioeconomic groups in mortality from other causes. The ratio of breast cancer survival in Finland between the highest and lowest social classes was 1.10 with corrected survival rates (censoring deaths from other causes) and 1.12 with relative survival rates (Karjalainen and Pukkala, 1990).

A third methodological issue concerns the exclusion from analysis of DCO cases, for which survival time is unknown. In this study the percentage of such cases was similar (8–9%) in all deprivation categories. We were able to estimate the effect of excluding DCO cases on observed survival (J Bullard, in preparation). As a ratio of the observed (unadjusted) survival at 5 years in the most affluent group, observed survival at 5 years in groups 2–5 was respectively 0.92, 0.87, 0.88 and 0.82. These ratios became 0.91, 0.86, 0.86 and 0.81 after correction for the exclusion of DCO cases, and their exclusion could thus have had very little effect on the gradient in survival reported here.

Fourth, the stage at diagnosis used in these analyses is not identical to the TNM stage. The key advantages are that, unlike TNM stage, it is available for most cases; it is simple; it has been assigned by Registry staff with a standard definition over many years; and, for cases for which both stage codes are available, it has almost identical prognostic significance (J-M Lutz, in preparation). It has been argued that the most important explanatory factor for socioeconomic variation in breast cancer survival is a difference in the stage distribution between deprivation categories, and in some studies deprived women have been shown to present at a more advanced stage than affluent women (Farley and Flannery, 1989; Karjalainen and Pukkala, 1990; Wells and Horm, 1992). No such pattern was observed in Scotland (Carnon *et al.*, 1994), or for younger women in this study. For older women, differences in the stage distribution did explain part of the variation in survival, the hazard ratio for the most deprived group falling from 1.68 to 1.47 after adjustment for stage.

Our results are similar to those from other studies in which survival differences between socioeconomic groups persisted after correction for the data available on stage at diagnosis (Dayal *et al.*, 1982; Bassett and Krieger, 1986; Karjalainen and Pukkala, 1990). Part of the gradient in survival by deprivation could be due to residual confounding by stage. If women from deprived areas were diagnosed less accurately than women from affluent areas, they would be understaged more often, leading to greater misclassification of stage at diagnosis in women from deprived areas. This assumption could not be tested with cancer registry data however.

Our findings suggest that special attention to early detection and rapid referral of breast cancer should be given to women aged 65 or more living in deprived areas. A strength of area-based analyses is that such women could be identified through their area of residence, perhaps for special health education programmes. The other prognostic factors that we studied had little impact (type of treatment) or no impact (morphology) on the gradient in survival by deprivation category.

We conclude that a gradient in breast cancer survival according to deprivation still existed after adjustment for stage at diagnosis, morphology and broad category of treatment. Other factors might be responsible for the observed gradient in breast cancer survival by deprivation category, such as a poorer host resistance among the deprived patients, which could be related to more co-morbidity, an adverse nutritional status, less social support and negative psychological factors, such as a lesser ability to cope with a cancer diagnosis. Aspects of the health care system which might be related to the lower survival of the lower socioeconomic groups are, apart from the type of treatment, adverse hospital referral patterns, the lower quality or appropriateness of treatment and worse compliance with treatment in these groups of patients. For most of these factors, however, information is not available from cancer registry records, and other approaches will be required to study their impact. Preliminary results from a study in our territory of breast cancer patients aged less than 50 suggest that survival was significantly affected by the use of adjuvant therapy (Wolfe *et al.*, 1993). Hospital referral patterns are being examined.

The Health of the Nation target for breast cancer envisages a 25% reduction in breast cancer mortality among women aged 50-69 by the year 2000. Improving the survival of breast cancer patients living in less affluent areas would make a substantial contribution to this target. In women aged 50-69 years the overall reduction would have been over 10% 5 years after diagnosis. Our results suggest that one way of achieving this improvement would be to focus on socioeconomic differences in stage at presentation in older women. In younger women, other factors, so far unidentified, are responsible for the socioeconomic gradient in breast cancer survival.

**Acknowledgements**
CTM Schrijvers was supported by a grant from the Research and Development programme of South West Thames Regional Health Authority.

**References**

ANSELL D, WHITMAN S, LIPTON R AND COOPER R. (1993). Race, income, and survival from breast cancer at two public hospitals. *Cancer*, **72**, 2974-2978.

BASSETT MT AND KRIEGER N. (1986). Social class and black-white differences in breast cancer survival. *Am. J. Public Health*, **76**, 1400-1403.

BONETT A, RODER D AND ESTERMAN A. (1984). Determinants of case survival for cancers of the lung, colon, breast and cervix in South Australia. *Med. J. Aust.*, **141**, 705-709.

CARNON AG, SSEMWOGERERE A, LAMONT DW, HOLE DJ, MALLON EA, GEORGE WD AND GILLIS CR. (1994). Relation between socioeconomic deprivation and pathological prognostic factors in women with breast cancer. *Br. Med. J.*, **309**, 1054-1057.

CARSTAIRS V AND MORRIS R. (1989). Deprivation and mortality: an alternative to social class? *Comm. Med.*, **11**, 210-219.

CARSTAIRS V AND MORRIS R. (1991). Deprivation and health in Scotland. Aberdeen University Press: Aberdeen.

CHAMBERLAIN J, MOSS SM, KIRKPATRICK AE, MICHELL M AND JOHNS L. (1993*a*). National Health Service breast screening programme results for 1991-2. *Br. Med. J.*, **307**, 353-356.

CHAMBERLAIN J, BOURNE HM AND THORNTON-JONES H. (1993*b*). UK, England, South Thames Region, 1983-87. In *Cancer Incidence in Five Continents*, Vol. VI, IARC Scientific Publications NO. 120, Parkin DM, Muir CS, Whelan SL, Gao Y-T, Ferlay J, Powell J. (eds) pp. 790-793. IARC: Lyon.

CHOUILLET AM, BELL CMJ AND HISCOX JG. (1994). Management of breast cancer in south-east England. *Br. Med. J.*, **308**, 168-171.

COLEMAN MP, ESTÈVE J, DAMIECKI P, ARSLAN A AND RENARD H. (1993). *Trends in Cancer Incidence and Mortality*. IARC Scientific Publications No. 121. IARC: Lyon.

CUTLER SJ AND EDERER F. (1958). Maximum utilisation of the life table method in analyzing survival. *J. Chronic Dis.*, **8**, 699-712.

DAYAL HH, POWER RN AND CHIU C. (1982). Race and socioeconomic status in survival from breast cancer. *J. Chronic Dis.*, **35**, 675-683.

DEPARTMENT OF HEALTH. (1992). *The Health of the Nation: a Strategy for Health in England*. HMSO: London.

DEPARTMENT OF HEALTH. (1993). *The Health of the Nation: Specification of National Indicators*. HMSO: London.

EXPERT ADVISORY GROUP ON CANCER TO THE CHIEF MEDICAL OFFICERS OF ENGLAND AND WALES. (1994). *Consultative Document: A Policy Framework for Commissioning Cancer Services*. Department of Health: London.

FARLEY TA AND FLANNERY JT. (1989). Late-stage diagnosis of breast cancer in women of lower socioeconomic status: public health implications. *Am. J. Public Health*, **79**, 1508-1512.

HAKULINEN T AND ABEYWICKRAMA KH. (1985). A computer program package for relative survival analysis. *Comp. Prog. Biomed.*, **19**, 197-207.

HAKULINEN T AND TENKANEN L. (1987). Regression analysis of relative survival rates. *Appl. Stat.*, **36**, 309-317.

KARJALAINEN S AND PUKKALA E. (1990). Social class as a prognostic factor in breast cancer survival. *Cancer*, **66**, 819-826.

KELSEY JL, GAMMON MD AND JOHN EM. (1993). Reproductive factors and breast cancer. *Epidemiol. Rev.*, **15**, 36-47.

KOGEVINAS M, MARMOT MG, FOX AJ AND GOLDBLATT PO. (1991). Socioeconomic differences in cancer survival. *J. Epidemiol. Commun. Health*, **45**, 216-219.

OPCS. (1987). *Mortality Statistics: Area, 1985*. Series DH5 no. 12. HMSO: London.

PAYNE CD. (1985). *The GLIM system release 3.77. Generalized Linear Interactive Modelling Manual*. Numerical Algorithms Group: Oxford.

SKEET RG. (1991). The Thames Cancer Registry. In *Cancer Registration: Principles and Methods*, IARC Scientific Publications No. 95, Jensen OM, Parkin DM, MacLennan R, Muir CS, Skeet RG. (eds). pp. 237-245. IARC: Lyon.

SKEET RG, THORNTON-JONES H AND MURRELLS TJ. (1987). UK, England, South Thames Region, 1978-1982. In *Cancer Incidence in Five Continents*, Vol. V, IARC Scientific Publications No. 88, Muir CS, Waterhouse JAH, Mack T, Powell J, Whelan SL (eds) pp. 664-667. IARC: Lyon.

THAMES CANCER REGISTRY. (1992*a*). *Cancer in South East Thames, 1987-1989: Cancer Incidence, Prevalence and Survival in Residents of the District Health Authorities in South East Thames*. Thames Cancer Registry: Sutton.

THAMES CANCER REGISTRY. (1992*b*). *Cancer in South West Thames 1987-1989: Cancer Incidence, Prevalence and Survival in Residents of the District Health Authorities in South West Thames*. Thames Cancer Registry: Sutton.

THAMES CANCER REGISTRY. (1994). *Cancer in South East England 1991: Cancer Incidence, Prevalence and Survival in Residents of the District Health Authorities in South East England*. Thames Cancer Registry: Sutton.

VÅGERÖ D AND PERSSON G. (1987). Cancer survival and social class in Sweden. *J. Epidemiol. Commun. Health*, **41**, 204-209.

WELLS BL AND HORM JW. (1992). Stage at diagnosis in breast cancer: race and socioeconomic factors. *Am. J. Public Health*, **82**, 1383-1385.

WOLFE CDA, BARTON J, BOURNE HM AND RICHARDS MA. (1993). Variation in the incidence and management of primary breast cancer in women under 50 years of age (abstract). *J. Epidemiol. Commun. Health*, **47**, 400.